# Relationship of Compositional, Mechanical, and Textural Properties of Gluten-Free Pasta Using Different Quinoa (*Chenopodium quinoa*) Varieties

**DOI:** 10.3390/foods9121849

**Published:** 2020-12-11

**Authors:** Jose Martin Ramos-Diaz, Tatjana Kince, Martins Sabovics, Göker Gürbüz, Asta Rauma, Anna-Maija Lampi, Vieno Piironen, Evita Straumite, Dace Klava, Kirsi Jouppila

**Affiliations:** 1 Department of Food and Nutrition, University of Helsinki, Agnes Sjöbergin Katu 2, FI-00014 Helsinki, Finland; goker.gurbuz@helsinki.fi (G.G.); asta.rauma@gmail.com (A.R.); Anna-Maija.Lampi@Helsinki.fi (A.-M.L.); Vieno.Piironen@helsinki.fi (V.P.); kirsi.jouppila@helsinki.fi (K.J.); 2 Faculty of Food Technology, Latvia University of Life Sciences and Technologies, Rigas Iela 22, LV-3001 Jelgava, Latvia; tatjana.kince@llu.lv (T.K.); martins.sabovics@llu.lv (M.S.); evita.straumite@llu.lv (E.S.); dace.klava@llu.lv (D.K.)

**Keywords:** quinoa, pasta, extrusion

## Abstract

Quinoa epitomizes the drive for healthier foods with ethnic concepts in developed countries, particularly among millennials. As a result, the popularity of quinoa as a gluten-free alternative has steadily grown over the last 20 years. Despite this, little is known about the impact of specific varieties on processed foods. The purpose of this study was to examine the impact of quinoa varieties (variety and content) on the mechanical and textural properties of buckwheat-based extruded pasta (spaghetti). Peruvian native (var. *rosada taraco*, *kuchivila*, *negra collana,* and *mistura*) and Latvian-grown (var. *titicaca*) varieties were independently incorporated to pasta between 5 and 20% (*w*/*w*). Pasta containing 20% quinoa var. *negra collana*, which presented the largest content of fiber and lowest content of saponin, was strongly associated to structural resilience (i.e., cohesiveness, firmness). Conversely, pasta containing 20% quinoa var. *Titicaca* appeared structurally weak (i.e., smooth). The addition of saponin-containing varieties to pasta (20%), such as *rosada taraco* and *mistura*, resulted in resilient structures with little effect on taste (incl. bitterness). Despite initial stability, pasta containing 20% quinoa var. *kuchivila* suffered heavy structural damage. In conclusion, the relationship of compositional, mechanical, and textural properties of pasta was strongly variety-dependent.

## 1. Introduction

Grocery shopping has gone through major changes over the last decade. The rise of millennials from a niche to a mass market seeking healthy, ethical, and locally produced food is setting up unprecedented challenges for food industry, particularly in advanced economies [1]. Quinoa (a grain endemic to the Andes of South America) probably owes its growing popularity to its formidable nutritional properties and millennial-appealing ethnic concept. Despite the agricultural interest to cultivate quinoa in the Northern Hemisphere, production fails to satisfy a growing demand. Thus, developing countries such as Peru and Bolivia still cultivate over 90% of quinoa consumed worldwide [2].

The regions around the Titicaca Lake are the world’s largest source of quinoa varieties, naturally adapted to various microclimates and soil conditions. Their resilience has been a matter of scientific investigation for various decades, but little is known about the technological potential of specific varieties. As a matter of fact, some authors [3,4] conducted variety-based studies describing quinoa as plainly as “black”, “white”, and “red”; insufficient for experimental reproducibility. 

Various studies which utilized quinoa as tested ingredient for pasta or noodles, observed the detrimental effect of quinoa on physicochemical and textural properties of the final product. For instance, Seol and Sim [4] incorporated up to 20% black quinoa to wheat-based noodles and observed that resilience reduced in inverse proportion to quinoa content. Schoenlechner et al. [5,6] found that the incorporation of up to 50% quinoa or amaranth to buckwheat-based fresh pasta resulted in a considerable increase of cooking losses and loss of firmness. These authors seemingly used Austrian quinoa, but varietal details were absent. Alternatively, various technological studies tend to assume that quinoa’s saponin content is negligible upon pearling [7,8], washing [9], or both [10].

Cold-forming wet extrusion is the most common industrial method for the production of fresh or dry pasta. The main advantages of this method includes high mixing capacity, flow production and control of feeding rates. Kahlon et al. [11] managed to produce extruded pasta from wholegrain quinoa flour with the incorporation of 5% guar gum (dry based). A consumer study revealed that, despite its moderate acceptability (61% respondents; *n* = 62), the quality of quinoa-based pasta required substantial improvement [11]. Cooking loss is indeed a key characteristic to assess the quality of pasta but, as previously reported [12], it may not necessarily correlate with consumer acceptability. Makdoud and Rosentrater [12] observed that, while their optimized quinoa-containing pasta (up to 50% quinoa) presented lower cooking losses (15–20%) than commercial counterparts (28%), quinoa-containing pasta was anyway stickier, softer, and subsequently less acceptable than expected (20% respondents would NOT eat the product again and 50% would PERHAPS eat it again). Most studies seem to revolve on quinoa’s technological hurdle, but little is discussed on quinoa’s varietal-based effects on pasta characteristics. 

The present study attempts to incorporate specific varieties of quinoa to buckwheat-based extruded pasta, and study the physicochemical, mechanical, and textural properties of the final product. The main target was not to optimize the production process, but to characterize the role of selected quinoa varieties in gluten-free egg-free spaghetti-type pasta with minimal additives.

## 2. Materials and Methods 

### 2.1. Raw Material

Quinoa grains belonging to the varieties *kuchivila* (K), *mistura* (M), *negra collana* (NC), and *rosada taraco* (RT) were cultivated and purchased from the district of Pomata, Region of Puno, Peru (16°19′12.00″ S; 69°13′48.00″ W; elevation, 3870 m). An additional quinoa sample belonging to the variety *titicaca* (T) was obtained from Cimbuli Ltd., district of Taurene, Region of Vidzeme, Latvia (57°10′12.00″ N; 25°37′12.00″ E; elevation, 244 m). 

### 2.2. Flour Preparation and Characterization

Quinoa grains were washed with water (1:10) at 40 °C using an Environmental Shaker–Incubator (ES-20, BIOSAN, Riga, Latvia) for 30 min at 250 rpm, and then dried in an airflow cabinet at 40 °C for 3 h. Grains were eventually milled using a laboratory mill PLM3100/B (Perten, Sweden). Commercial buckwheat flour (Hercogs ^®^, Rigas Dzirnavnieks Ltd., Riga, Latvia) was purchased from local market. A commercial mixture of stabilizers (Guar gum E412 and Xanthan gum E 415) was purchased from Palsgaard ^®^ (Hedensted, Denmark). The chemical composition (except for saponin content) was determined by following methods described by the American Association of Cereal Chemists (AACC) [13], while the particle diameters of flours were measured via a laser diffraction particle size analyzer (Mastersizer Hydro 3000 SM, Malvern Instruments Ltd., Worcestershire, UK); the particle refractive index was set at 1.46. 

### 2.3. Saponin Analysis 

Saponin was extracted from pre-treated ground samples (fat removed with heptane) via accelerated solvent extraction (ASE^®^) using ethanol–water mixture (80:20, *v*/*v*). Aglycones (oleanolic acid, hederagenin, serjanic acid, and phytolaccagenic acid) were subsequently liberated by acid hydrolysis and derivatized to trimethyl silyl ethers. Identification of individual aglycones was done by gas chromatography mass spectrometry, and their quantification done by flame ionization detector using cholesteryl decanoate (>99%, Nu-Chek-Prep, Inc., Elysian, MN, USA) as internal standard [14,15,16]. In the end, total saponin contents were expressed as the sum (mg/g of solids) of the afore-mentioned aglycones. 

### 2.4. Pasta Extrusion 

**Figure 1:**
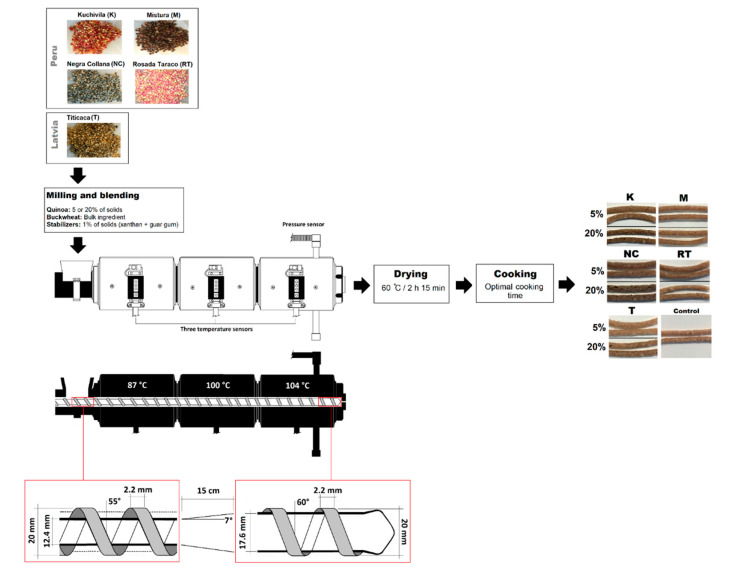
Flow chart for pasta production and technical description of the extruder (D = 20 mm, L/D 28:1). Sites for temperature/pressure monitoring are shown.

Buckwheat-based pasta changing in variety (*kuchivila*, *mistura*, *negra collana*, *rosada taraco,* and *titicaca*) and content of quinoa (5 and 20% of solids) according to a 5 × 2 factorial design was prepared under the same extrusion conditions; the control sample was entirely made of buckwheat (100% of solids). Mixture of stabilizers was added at 1% of solids. Extruded pasta was prepared using a single-screw extruder (PCE Extrusiometer L–Series, Göttfert, Germany) that consisted of three sections. The temperature profile was fixed at 87 °C (Section 1), 100 °C (Section 2), and 104 °C (Section 3). Screw speed, feeding rate and water content of dough were set at 25 rpm, 25 g/min and 55%, respectively. Upon extrusion, samples were dried in a convection oven at 60 °C for 2 h and 15 min (Figure 1).

### 2.5. Determination of Physicochemical and Mechanical Properties 

Optimal cooking time (OCT) was calculated as the minimum time needed for the core of a pasta strand to be fully cooked in boiled water. Twenty strands of pasta (4 cm in length) were submerged in boiling water (time zero) and collected, one-by-one, every 30 s [13]. The collected samples were pressed between two Petri dishes for qualitative assessment. Optimally cooked pasta allowed full pressing and observable homogenous distribution against a light source. Cooking loss was calculated as the percentage of solids lost in boiling water during the optimal cooking of pasta. Five strands of pasta (4 cm in length) were placed in boiling water and optimally cooked (OCT). Cooked pasta strands were removed, and the beaker containing the cooking water placed into an airflow oven at 105 °C for 12 h. The loss of pasta during cooking was calculated by weight difference using Equation (1).

(1) $$\%\;Cooking\;loss\;=\;\frac{\left[\left(W_{Bs}\right)-\left(W_{B}\right)\right]\times 100}{\left(W_{p}\right)}$$

where *W_BS_* is the weight of the beaker containing dried remnants of the cooked pasta, *W_B_* is the weight of the empty beaker, and *W_P_* is the weight of the dried pasta sample prior to cooking. 

Mechanical firmness (M-Firmness) was measured as the positive area (N.mm) under a force–distance curve when compression was perpendicularly enforced on five strands of pasta. Mechanical stickiness (M-Stickiness) was measured as the negative area (N.mm) under a force–distance curve plotted upon the retraction of the probe. Mechanical hardness (M-Hardness) was the peak force resulting from the perpendicular compression of five strands of pasta. A Texture Analyzer (TA-XT2, Stable Micro Systems Ltd., Surrey, UK) was fitted with a bladed rectangular-shaped aluminum probe (90 × 69 × 3 mm). Pasta samples were placed perpendicularly over a sample holder (slit width, 3.5 mm). The speed of the crosshead was 10.2 mm/min. Pasta samples were optimally cooked, and then placed in cold water for 5 min prior to analysis. Colour of pasta samples was determined by using a Tristimulus colorimeter Colour Tec PCM/PSM (Accuracy Microsensors, Inc., New York, NY, USA). The CIE Lab colour values included L* (lightness–darkness), a* (redness-greenness), b* (yellowness-blueness). The total colour difference (*ΔE*) between control (100% buckwheat) and quinoa-containing pasta was calculated using the Equation (2).

(2) $$∆E=\;\sqrt{{\left(L_{c}-L_{p}\right)}^{2}+{\left(a_{c}-a_{p}\right)}^{2}+\;{\left(b_{c}-b_{p}\right)}^{2}}$$

where *L_c_, a_c_* and *b_c_*, and *L_p_*, *a_p_* and *b_p_* are the corresponding values for the control and quinoa-containing pasta.

### 2.6. Stereomicroscopy Imaging 

Dried pasta samples were cooked in accordance with their OCTs. Pasta was then placed in deionized water and stored at 5 °C for 24 h. By using a knife blade, a strand of pasta was sliced into thin sheets and placed in the sample holder of a Stemi DV4 stereomicroscope (Carl Zeiss MicroImaging, Göttingen, Germany). Images were processed using Zen software (6.3 × digital zoom). The average diameter of ten images was calculated.

### 2.7. Sensory Evaluation

#### 2.7.1. Assessors

The sensory panel (*n* = 7, 6 females, 1 male, aged 30–50 years) was recruited from the Latvia University of Life Sciences and Technologies (staff). Training and evaluation sessions were held in English at the sensory laboratory of the Faculty of Food Technology, in accordance with the guidelines described in ISO 8586 [17]. 

#### 2.7.2. Sensory Profiling 

Training was performed to familiarize the sensory panel with gluten-free pasta (spaghetti type or similar) and develop a set of descriptors, reference samples, and definitions. Initially, assessors were presented with various commercial pasta products to generate a preliminary list of descriptors linked to texture and taste. The panel eventually agreed upon a list of reference samples and their corresponding definitions. Training in sensory profiling lasted for up to 12 h.

Each assessor evaluated 11 samples (including control) in duplicate (30-min break between sessions). Each sample consisted of three strands of pasta (10 cm in length), presented in a plastic-foil-covered porcelain dish. Samples were coded and presented to each assessor in random order. Sensory evaluation was conducted in individual booths at room temperature. Assessors removed the foil and rated the intensity of the descriptors on structured 10-cm line scales with anchors: 1 (not at all) and 10 (very). Textural attributes were cohesiveness, firmness, grainy, smoothness and stickiness while taste attributes were bitterness and overall taste. Assessors were provided with water to cleanse their palates between samples. 

### 2.8. Statistical Analysis 

The effect of the quinoa variety (*kuchivila*, *mistura*, *negra collana*, *rosada taraco,* and *titicaca*), content of quinoa (0, 5, and 20% of solids) and their interaction on the sensory attributes of cooked pasta was statistically analysed by two-way repeated-measures analysis of variance (ANOVA) in SPSS (SPSS 25.0, PASW Statistics, Chicago, IL, USA). The statistically significant effect (*p* < 0.05) of variety and content of quinoa on the perception of samples was investigated according to a 5 × 3 × 2 factorial design (sensory evaluation conducted in duplicate). The potential interaction of replicates would indicate inconsistency in sensory ratings. Ratings on texture and taste were combined with compositional, mechanical, and physicochemical measurements through principal component analysis (PCA); this allowed us to observe correlations among samples and define key characteristics (The Unscrambler Version 10.5.1; CAMO Software AS, Oslo, Norway). 

Statistical comparisons within chemical, physicochemical, and/or structural values were performed via one-way ANOVA (*p* < 0.05) using LSD (least-significant difference) at level p of 5%. Analyses were conducted in triplicate except for the determination of fat (duplicate), fiber (six replicates), strand diameter (seven replicates), mechanical properties (nine replicates), and colour (ten replicates). 

## 3. Results and Discussions

### 3.1. Characterization of Flours

Quinoa flours (regardless of the variety) had considerably higher content of protein, fiber, fat, and ash compared to buckwheat flour (bulk ingredient). However, differences were also observed among quinoa flours. T and NC presented the highest contents of protein (almost 16% of solids) while RT presented one third less (around 11% of solids). In terms of fiber, NC presented the highest content (around 20% of solids) while T presented almost half of it. The contents of fat and ash are roughly similar among all quinoa flours (around 7% and 4% of solids, respectively). Further details on individual flours and the calculated composition of flour mixtures are found in Table 1. According to Quinto et al. [18], the content of protein and fiber in NC was just 11.2% and 3.6% of solids, respectively. This is remarkably low compared to our results and other studies [19,20]. Reguera et al. [20] observed that quinoa var. *titicaca* from Chile had consistently more protein than the one from Spain (18% > 15%). Similarly, Pulvento et al. [21] found that quinoa var. *titicaca* from Italy had between 14 and 17% protein, on average lower than the Chilean quinoa. In the present study, quinoa var. *titicaca* from Latvia (i.e., T) had protein content comparable to the ones from Spain and Italy (Table 1). The content of saponin (after washing) was the highest in RT and M whereas NC presented negligible amounts (Table 1). The latter agrees with the results published by Escribano et al. [22] where quinoa var. *negra collana* had among the lowest contents of saponin compared to 28 other varieties. According to Pulvento et al. [21], the content of saponin in raw quinoa var. *titicaca* varied between 6 and 17 mg/g of solids. These values are understandably higher than the one in the present study (2.7 mg/g of solids) where cleaning took place. 

Despite similar pre-treatment and milling, particle sizes of quinoa flours showed considerable variation in terms of surface area moment mean or D[3,2] and volume moment mean or D[3,4] (Table 1). M had the largest D[2,3] and D[4,3] while NC presented nearly half of them. Buckwheat flour presented the largest particle size (i.e., D[3,2]) compared to quinoa flours. Interestingly, similar studies [6,12] have utilized flours with particles sizes between 250 and 700 µm, considerably larger than those tested in the present study (from 112 µm to 221 µm).

### 3.2. Properties of Extruded Pasta

#### 3.2.1. Large-Scale Structures

**Figure 2:**
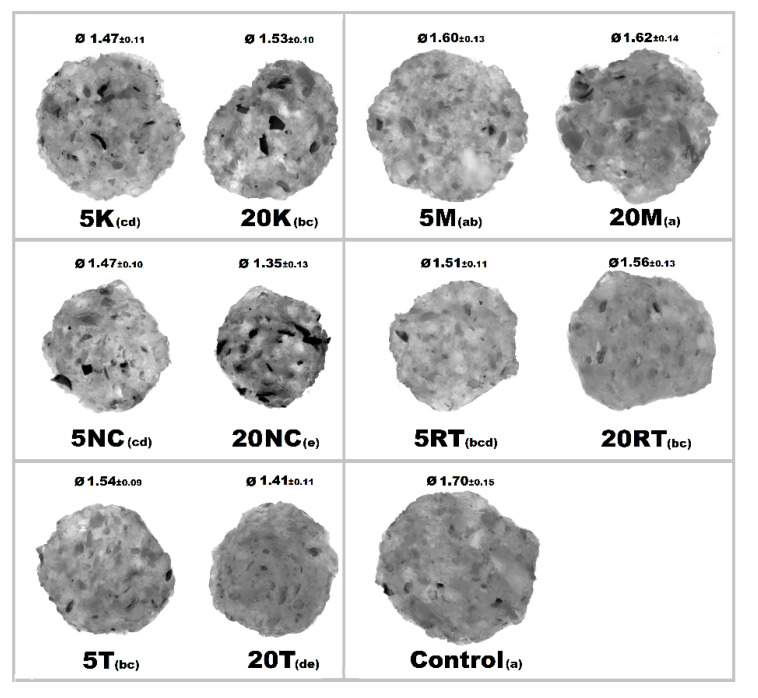
Cross-sectional images of cooked pasta strands containing 5 and 20% quinoa var. *kuchivila* (5 K, 20 K), *mistura* (5 M, 20 M), *negra collana* (5 NC, 20 NC), *rosada taraco* (5 RT, 20 RT), and *titicaca* (5 T, 20 T). These samples were obtained under the following conditions. Temperature profile: 87, 100, and 104 °C; Screw speed: 25 rpm; Water content of dough, 55%; Oven drying, 60 °C/2.25 h. Diameter of cooked pasta strands (Ø, mm) and the corresponding SD are shown. Raw data in Suplementary Materials (Table S1). Different letters indicate significant difference at level *p* of 5%.

There were observable diametric variations between quinoa-containing pasta and the control (Figure 2; Table S1). Most of quinoa-containing pasta (20 RT > 5 T > 20 K > 5 RT > 5 K > 5 NC > 20 T > 20 NC) presented smaller diameter than control, while the rest (20 M > 5 M) showed no statistical difference to control. Among quinoa-containing pasta, 20 NC presented by far the smallest diameter whereas 20 M presented the largest one. Caperuto et al. [23] noticed that increasing contents of quinoa reduced the volume of egg-containing corn-based pasta. Similarly, Bouasla et al. [24] reported that the addition of fiber-containing legumes (yellow pea, chickpea, and lentil) had a detrimental effect on the expansion ratio of rice-based pasta. Visually, pasta containing RT and T were the most similar to control. In both cases, the presence of grain hull was minimally perceivable upon inspection while pasta containing K and NC presented fragments of grain hull spread across its cross-sectional area (Figure 2). Possibly, this had a detrimental effect on the diametric size of the pasta (e.g., 20 NC).

#### 3.2.2. Mechanical and Physicochemical Properties

Around half of quinoa-containing pasta (5 TL > 20 RT > 20 TL > 5 RT > 5 K > 5 NC) had higher M-firmness and M-hardness than control. Generally, quinoa-containing pasta seemed less sticky than control (Table 2). The increasing content (from 5 to 20% of solids) of K, T and—to a lesser extent—NC appeared to reduce M-firmness and M-hardness while M and RT showed the opposite. Makdoud and Rosentrater [12] found that increasing quinoa (from 10 to 50% of solids) had little effect on the Young’s modulus and toughness of rice-based pasta containing amaranth and egg. Larusso et al. [25] replaced 20% of durum wheat semolina with quinoa flour, leading to structural hardening. In the present study, the greater incorporation of quinoa varieties with larger content of fiber and protein (like K and NC) seemed to weaken pasta structures while those with lesser contents of protein and fiber (like M and RT) strengthened it. Petitot et al. [26] explained that the presence of fiber fractions might lead to cracks inside the pasta strand thereby weakening their structure.

While quinoa-containing pasta was generally darker than control, those containing 20 M and 20RT were lighter. Only, 5 RT and—to a lesser extent—20 NC displayed comparatively higher redness than control. Statistically, the addition of quinoa increased the yellowness of pasta, except for NC. Overall, 20 T, 20 K and 20 NC showed the largest colour differences (in decreasing order) compared to control *(*∆E; Table 2). These results agree with those of Larusso et al. [25], where quinoa darkened, yet mildly strengthened the red colour of semolina-based pasta. Another study [5] showed that black quinoa (up to 20%) darkened wheat-based noodles and reduced redness and yellowness. Clearly, the testing of different quinoa varieties resulted in a wide range of colour tonalities and shades. However, drastic changes were not observed.

**Figure 3:**
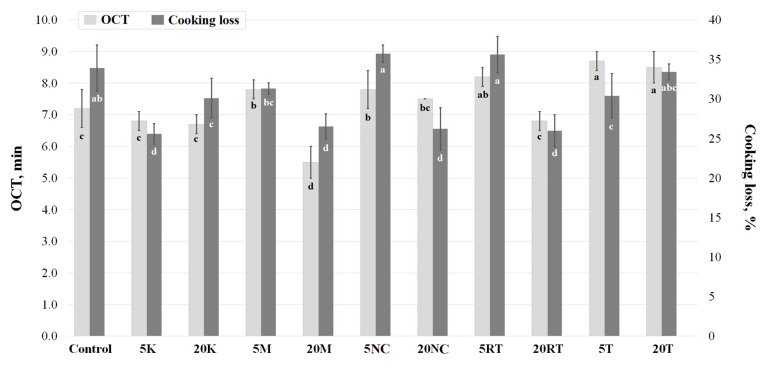
Optimal cooking time (OCT) and cooking loss of buckwheat-based pasta containing 5 or 20% quinoa varieties. Different letters indicate significant difference at level *p* of 5%. Corresponding dataset in Supplementary Materials (Table S2). Error bar shows ± standard deviation.

The incorporation of T increased OCT while the incorporation of M decreased it. Pasta containing M and RT showed a slight increase in OCT (5 M and 5 RT) before falling (20 M and 20 RT) by around one third (Figure 3; Table S2). Mastromatteo et al. [27] used a sensory panel to determine the OCT of all maize-based quinoa-containing pasta samples, and reached a consensus at 7 min. In the present study, the OCT varied between 5 and 9 min, and seemed to be inversely proportional to the content of quinoa. This agrees with Chillo et al. [28] where the incorporation of quinoa to semolina-based pasta reduced sensory-based OCT from 8 to around 5 min. Sosa et al. [29] speculated that shorter OCT values could respond to a greater water accessibility through cracked non-continuous pasta surface.

The initial incorporation (5%) of M, NC and RT seemed to maintain or increase the cooking losses, but greater addition (20%) led to a better retention of solids. Conversely, the initial incorporation of K and T showed greater retention of solids while further addition reverted to losses comparable to control (Figure 3). Evidently, cooking losses correlated strongly with OCT; the longer time pasta stayed in boiling water, the more leaching took place. It is difficult to establish a relationship, but it seemed that, except for NC, varieties with the largest contents of saponin (e.g., RT and M) were involved in greater retention of solids. Böttcher and Drusch [30] explained that saponin (particularly those with triterpenoid structure like in quinoa) is likely to form viscoelastic films and stable emulsions.

### 3.3. Sensory Evaluation

**Figure 4:**
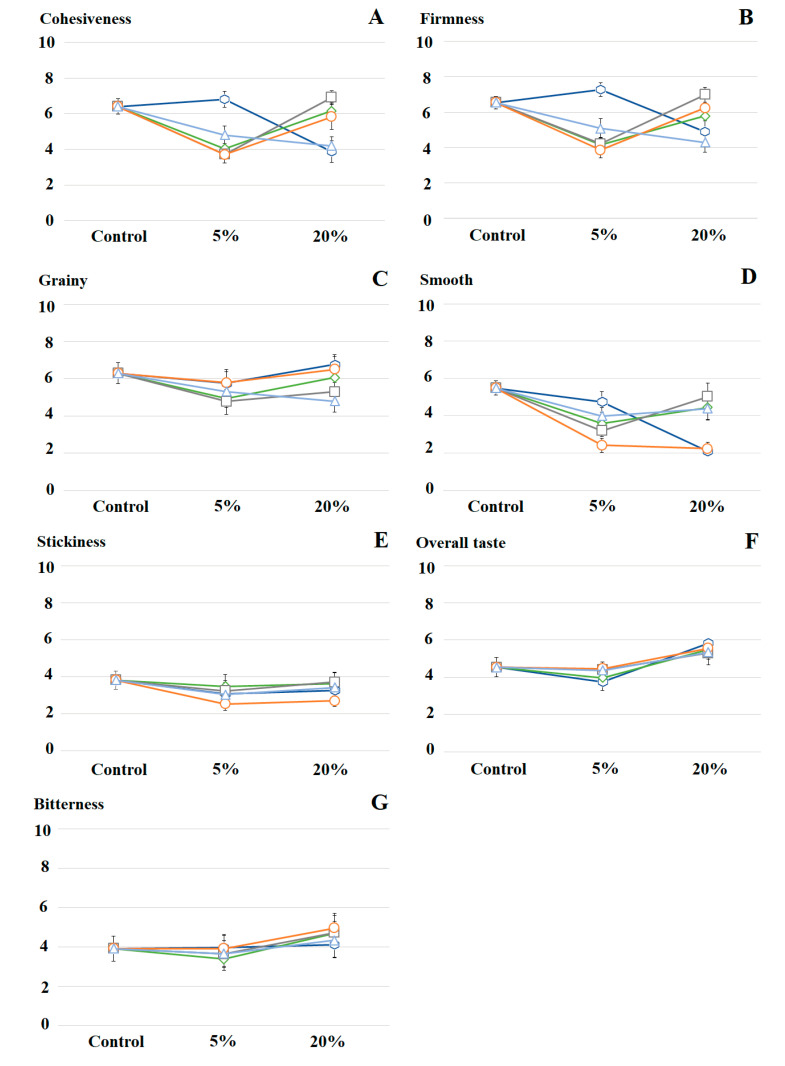
Sensory profiles of buckwheat-based pasta containing quinoa var. *kuchivila* (blue, **⬡**), *mistura* (green, **◊**), *rosada taraco* (gray, **▢**), *negra collana* (orange, **○**), *titicaca* (light blue, △). Sensory attributes tested: Cohesiveness (**A**), firmness (B), grainy (**C**), smooth (**D**), stickiness (**E**), overall taste (**F**) and bitterness (**G**). Corresponding dataset and raw data in Suplementary Materials (Tables S3 and S4). Error bar shows ± standard error.

Based on the attributes and evaluation techniques listed in Table 3, it was observed that quinoa-containing pasta showed considerable differences associated to perceivable texture rather than to taste (Figure 4; Tables S3 and S4). Changes in the content and variety of quinoa had combined effect on the cohesiveness of pasta (interaction, F(8,40) = 14.4, *p* < 0.0001). Increasing contents of K and T reduced consistently the cohesiveness of pasta, while the initial incorporation (5%) of RT, M, and NC led to a pronounced decrease of cohesiveness, and eventual rise at 20% (Figure 4A). Regarding firmness, changes in the content and variety of quinoa had individual and combined effect on pasta (main effect of content, F(2,10) = 22.1, *p* < 0.0001; main effect of variety, F(4,20) = 5.8, *p* < 0.003; interaction effect, F(8,40) = 11.7, *p* < 0.001). The addition of K and T reduced steadily the firmness of pasta, while the addition of RT, M, and NC initially reduced firmness, and eventually raise it at 20% (Figure 4B). Accordingly, Mastromatteo et al. [27] reported an increase in firmness upon the addition of quinoa (from 7.5% to 22.5%) to maize-based pasta.

This also supported the findings of Wu et al. [31], who noticed a direct relation between cohesiveness and the protein content of quinoa. Probably, the most distinct feature of pasta containing M and RT was their higher content of saponin, which may have vaguely affected the perceivable firmness of the final product. Once again, this argument loses strength if we include the high-protein high-fiber but saponin-free NC. It is reasonable to suspect that quinoa starch, along with protein and saponin, could be playing a pivotal role in the development of textural characteristics. Unfortunately, little is known about starch from these particular varieties.

Apparently, changes in the variety of quinoa altered the perceived graininess of pasta (main effect, F(4,20) = 6.6, *p* < 0.001) while the content of quinoa had no perceivable effect on graininess. Pasta containing K and NC were consistently perceived the grainiest while pasta containing T were rated the least grainy (Figure 4C). Regarding smoothness, changes in variety and content of quinoa had individual and combined effect on pasta (main effect of variety, F(4,20) = 9.6, *p* < 0.0001; main effect of content, F(2,10) = 9.6, *p* < 0.005; interaction effect, F(8,40) = 9.8, *p* < 0.0001). Increasing content of K and NC in pasta reduced consistently its perception of smoothness whereas the addition of M, T and RT slightly reduced smoothness at 5%, but increased it at 20% (Figure 4D). Seemingly, insoluble fiber from K and NC increased the perception of graininess and roughness (as opposite to smoothness). This phenomenon has been reported in previous studies [24,32]. Changes in variety and content of quinoa had minimal effect on the stickiness (Figure 4E).

When it comes to perceived overall taste and bitterness—despite the complexity associated to the study of "bitterness" [33]—the incorporation of up to 20% quinoa had little effect on pasta (Figure 4F,G). This goes in line with the results of Mastromatteo et al. [27], where the incorporation of up to 22.5% quinoa to maize-based pasta had no statistical effect on the overall taste. Similarly, Demir and Bilgicli [34] noticed minimal changes in the perceivable taste of rice–corn-based pasta containing up to 20% quinoa (raw or germinated).

### 3.4. Principal Component Analysis

**Figure 5:**
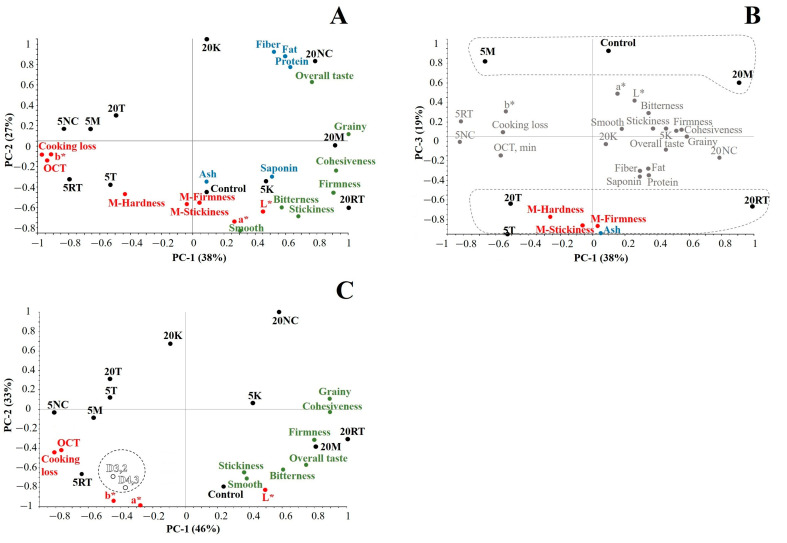
Principal component analysis bi-plots for compositional, physicochemical, mechanical, and sensory properties (**A**,**B**) accounting for a total variance of 84% (PC-1, PC-2, and PC-3). Principal component analysis bi-plots for physicochemical and sensory properties (**C**) with emphasis on particle diameters (D3,2: Surface area moment mean; D4,3: volume moment mean); this plot accounts for a total variance of 79% (PC-1 and PC-2). Factor scores: 5% quinoa var. *kuchivila* (5 K), 20% quinoa var. *kuchivila* (20 K), 5% quinoa var. *mistura* (5 M), 20% quinoa var. *mistura* (20 M), 5% quinoa var. *negra collana* (5 NC), 20% quinoa var. *negra collana* (20 NC), 5% quinoa var. *rosada taraco* (5 RT), 20% quinoa var. *rosada taraco* (20 RT), 5% quinoa var. *titicaca* (5 T), 20% quinoa var. *titicaca* (20 T), 100% buckwheat (control). Factor loadings for physicochemical and mechanical properties (RED): a* (green-red colour component); b* (blue-yellow colour component); Cooking loss; L* (lightness); M-Firmness (firmness of cooked pasta measured through mechanical methods); M-Hardness (hardness of cooked pasta measured through mechanical methods); M-Stickiness (stickiness of cooked pasta measured through mechanical methods); OCT (Optimal cooking time). Factor loadings for sensory properties (GREEN): Cohesiveness; Firmness; Grainy; Smooth; Stickiness; Overall taste; Bitterness. Factor loadings for compositional characteristics: Ash; Fat; Fiber; Protein; Saponin.

Changes in variety and content of quinoa (compositional alteration) seemed to alter physicochemical, mechanical, and textural properties of buckwheat-based pasta. For instance, 5 NC, 5 M, and 5 RT were strongly associated to less desirable characteristics such as high cooking losses, high OCT and low cohesiveness, among others, while 20 NC, 20 M, and 20 RT showed mostly desirable characteristics such as low cooking losses, low OCT and high cohesiveness (Figure 5A). The inevitable increase of fiber, fat, protein, and saponin seemed to contribute (in one way or another) to the structural stability and sturdiness of pasta. This may result counter-intuitive given the weakening role of insoluble fiber on solid/semisolid food systems [23,30]. This was probably the case for pasta containing K where 5 K was linked to, e.g., high cohesiveness and low cooking losses whereas 20 K presented considerably weaker structure. 5 T and 20 T showed consistently the least desirable characteristics associated to texture (except for low graininess).

Generally, the mechanical and textural properties of buckwheat-based pasta containing quinoa showed weak correlation; 20 RT and (to a lesser extent) 5 K are probably the only exceptions. 20RT and 5K showed high firmness and stickiness via textural or mechanical (M-) measurements (Figure 5A,B). Mechanically speaking, 5T was consistently associated with M-hardness, M-stickiness, and M-firmness (Table 1) while 20 NC, 20 K, 5 M, and 20 M showed mechanical weakness. Control was assessed as mechanically weak and the incorporation of quinoa seemed to have improved the technological characteristics of buckwheat-based pasta. On the other hand, larger particle size of flours was associated to less desirable technological characteristics such as low cohesiveness, low firmness, high OCT, and high cooking losses (Figure 5C).

Even though drastic changes in overall taste and bitterness were not observed across samples, results allowed us to infer a progressive increase in their perception (from 5% to 20% quinoa). From all quinoa samples, NC and RT seemed to elicit the strongest taste response. 20 NC was surprisingly linked to overall taste and bitterness—despite having negligible amounts of saponin—while 20 RT was only associated to bitterness (Figure 5A). Major differences were expected given the 10-fold difference in their contents of saponin (20 RT > 20 NC, Table 1).

## 4. Conclusions

This study shows that the mechanical, textural, and compositional characteristics of buckwheat-based pasta may change depending on the tested quinoa varieties. In general, the addition of RT, NC, and M seemed to show a modest increase in the desirable (technological) characteristics of pasta, while T and K showed the opposite effect. Pasta containing varieties with the highest content of insoluble fiber, like NC and K, presented the lowest cross-sectional diameter. The incorporation of NC, M, and R reduced OCT and, subsequently, cooking losses. The perception of textural resilience (e.g., firmness and cohesiveness) was strongly associated to the incorporation of RT, M, and NC, while the opposite was observed with K and, particularly, T. Despite the apparent incongruity, mechanical and textural results seemed to agree on the strengthening role of RT. This variety had among the lowest content of protein, fiber, and fat, but the largest content of saponin. In the present study, there was no observable relationship between taste and saponin. Understanding the dynamics between starch, protein and saponin may shed light on the mechanical and textural behaviour of quinoa-containing products. This is an important step towards the selection of naturally-existing varieties with promising characteristic for product development.

## Figures and Tables

**Table 1:** Chemical composition and particle diameters of quinoa varieties and buckwheat flours. The calculated values of flour blends containing 5 or 20% quinoa varieties were also included. Error shows ± standard deviation.

		Content (g/100 g d.m.)						
Variety	Moisture, %	Protein, %	Fiber, %	Fat *, %	Ash, %	Total Saponin Content, mg/g d.m.	D[3,2], μm	D[4,3], μm
Kuchivila (K)	12.3 ^b^ ± 0.7	13.9 ^b^ ± 0.1	18.8 ^b^ ± 1.5	7.0	3.9 ^ab^ ± 0.2	1.9 ^c^ ± 0.1	27.9 ^d^ ± 0.8	180 ^b^ ± 8.5
5 K		11.0	3.4	3.5	1.6	0.10	81.5	219.6
20 K		11.4	5.8	4.1	1.4	0.38	73.0	213.4
Mistura (M)	11.2 ^c^ ± 0.6	12.7 ^c^ ± 0.1	12.5 ^c^ ± 0.9	6.6	3.6 ^bc^ ± 0.1	3.4 ^a^ ± 0.1	35.4 ^b^ ± 0.4	218 ^a^ ± 7.0
5 M		10.9	3.1	3.5	1.5	0.17	81.9	221.5
20 M		11.2	4.5	4.0	1.4	0.68	74.5	221.0
Negra collana (NC)	10.8 ^c^±0.6	15.6 ^a^ ± 0.1	20 ^a^ ± 1.3	6.7	3.1 ^d^ ± 0.1	0.34 ^d^ ± 0.02	20.3 ^e^ ± 0.2	112 ^d^ ± 4.3
5 NC		11.1	3.4	3.5	1.5	0.02	81.1	216.2
20 NC		11.8	6.0	4.0	1.4	0.07	71.5	199.8
Rosada taraco (RT)	9.6 ^d^ ± 0.5	11.4 ^d^ ± 0.05	11 ^d^ ± 0.4	6.4	3.2 ^cd^ ± 0.3	3.4 ^a^ ± 0.2	29.6 ^d^ ± 0.4	166 ^c^ ± 4.1
5 RT		10.9	3.0	3.5	1.5	0.17	81.6	218.9
20 RT		10.9	4.3	3.9	1.4	0.68	73.4	210.6
Titicaca (T)	14.9 ^a^ ± 0.3	15.9 ^a^ ± 0.2	9.5 ^e^ ± 0.4	7.3	4 ^a^ ± 0.1	2.7 ^b^ ± 0.2	31.4 ^c^ ± 0.5	185 ^b^ ± 7
5 T		11.1	2.9	3.5	1.6	0.14	81.7	219.9
20 T		11.8	4.0	4.1	1.4	0.54	73.7	214.4
Buckwheat (control)	8.5 ^e^ ± 0.2	10.8^e^ ± 0.5	2.6 ^f^ ± 0.3	3.3	1.4 ^e^ ± 0.4	n.d. **	84.3 ^a^ ± 2.8	221.7 ^a^ ± 0.6

* Analyses were conducted in duplicate; ** Total saponin content in buckwheat flour was not determined and assumed cero for calculations. Different letters indicate significant difference at level *p* of 5%.

**Table 2:** Mechanical properties and colour characteristics of buckwheat-based pasta containing 5 or 20% quinoa varieties. Error shows ± standard deviation.

Variety	M-Firmness, N.mm	M-Hardness, N	M-Stickiness, N.mm	L*	a*	b*	*ΔE*
Kuchivila (K)							
5K	7.2 ^b^ ± 1.4	2.9 ^bc^ ± 0.6	0.7 ^b^ ± 0.1	65.6 ^c^ ± 1.3	3 ^cd^ ± 1.4	16.1 ^abc^ ± 1.8	3 ^de^ ± 1.6
20K	4.7 ^de^ ± 1.1	1.8 ^d^ ± 0.5	0.1 ^e^ ± 0.03	59.3 ^g^ ± 1	2.7 ^de^ ± 0.1	16.8 ^ab^ ± 0.7	7.9 ^a^ ± 1.7
Mistura (M)							
5 M	3.8 ^e^ ± 0.5	1.5 ^d^ ± 0.2	0.1 ^e^ ± 0.05	63.4 ^e^ ± 0.9	3.8 ^b c^ ± 1.1	16.8 ^ab^ ± 2.4	4.3 ^cd^ ± 1.1
20 M	5.3 ^d^ ± 1.4	1.9 ^d^ ± 0.6	0.3 ^cd^ ± 0.1	68.7 ^a^ ± 0.8	3.3 ^c d^ ± 2	17.2 ^a^ ± 1.8	3.6 ^cde^ ± 1.3
Negra collana (NC)							
5 NC	7.1 ^bc^ ± 1.6	2.9 ^bc^ ± 0.8	0.3 ^cd^ ± 0.1	64.5 ^d^ ± 0.6	2.4 ^e^ ± 0.8	15.8 ^bc^ ± 0.7	3.6 ^de^ ± 1.9
20 NC	5.7^cd^ ± 0.9	2.2 ^cd^ ± 0.3	0.2 ^de^ ± 0.1	62 ^f^ ± 0.7	4.8 ^ab^ ± 0.8	12 ^d^ ± 1.4	6.1 ^b^ ± 1.6
Rosada taraco (RT)							
5RT	7 ^bc^ ± 2.1	3.1 ^b^ ± 1.2	0.4 ^c^ ± 0.2	67.5 ^b^ ± 1.1	5 ^a^ ± 1.4	15.1 ^c^ ± 1.4	2.5 ^e^ ± 1.1
20RT	9.3 ^a^ ± 1.7	4.2 ^a^ ± 1	0.9 ^a^ ± 0.3	68.7 ^a^ ± 1.5	2.7 ^e^ ± 1.2	16.2 ^abc^ ± 1.7	4.1 ^cd^ ± 1.6
Titicaca (T)							
5T	10.4 ^a^ ± 2.5	4.9 ^a^ ± 1.6	0.6 ^b^ ± 0.3	62.3 ^f^ ± 1.1	3.1 ^cd^ ± 1.2	15.6 ^bc^ ± 1	5.2 ^bc^ ± 1.7
20T	7.3 ^b^ ± 2.2	3.2 ^b^ ± 0.9	0.4 ^c^ ± 0.2	59.3 ^g^ ± 0.6	1.9 ^e^ ± 0.5	16.7 ^ab^ ± 0.6	8.1 ^a^ ± 2.1
Buckwheat (control)	4.8 ^de^ ± 0.9	1.9 ^d^ ± 0.5	0.6 ^b^ ± 0.1	66.8 ^b^ ± 1.5	3.8 ^bc^ ± 0.8	15.1 ^c^ ± 1.6	

Different letters indicate significant difference at level *p* of 5%.

**Table 3:** Attributes, evaluation techniques, and references used for sensory profiling of buckwheat-based pasta.

Attribute	Evaluation Techniques	Reference			
		Not at All		Very	
Cohesiveness	Place a strand of pasta in your mouth. Evaluate the time it takes to bite it through	Pasta S3 **		Pasta *Mega di cato*^®^	
		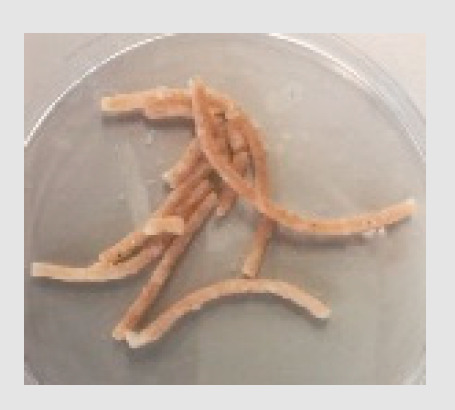	OCT: 7 min RIT: 2 min RRT: 5 min	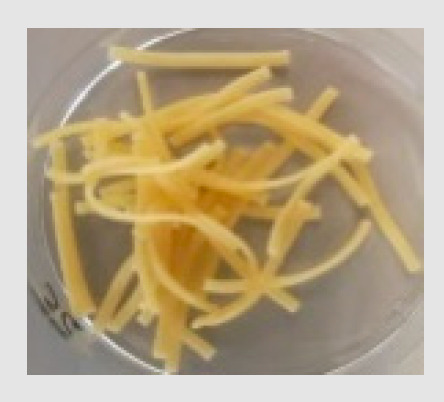	OCT: 4 min RIT: 0 min RRT: 5 min
Firmness	Place a strand of pasta between your front teeth. Evaluate the force needed to break it	Pasta S3 **		Pasta *Mega di cato*^®^	
		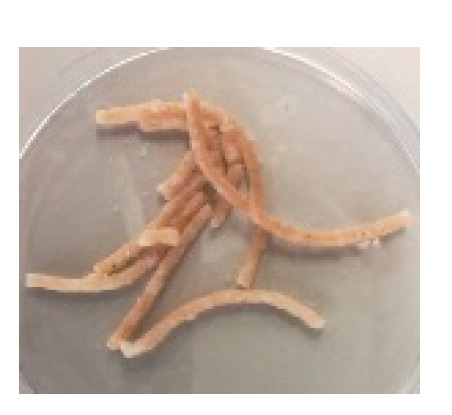	OCT: 7 min RIT: 2 min RRT: 5 min	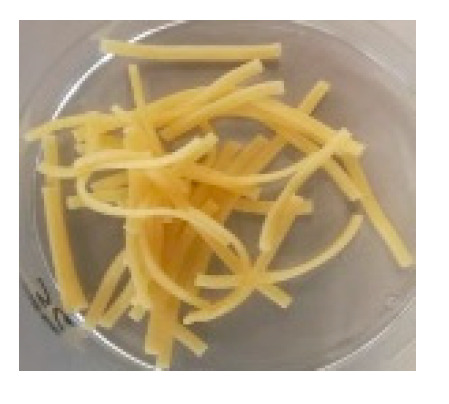	OCT: 4 min RIT: 0 min RRT: 5 min
Grainy	Place a strand of pasta in your mouth. Evaluate the coarseness by rubbing it between your tongue and palate	Pasta *Mega di cato*^®^		Pasta S1 *	
		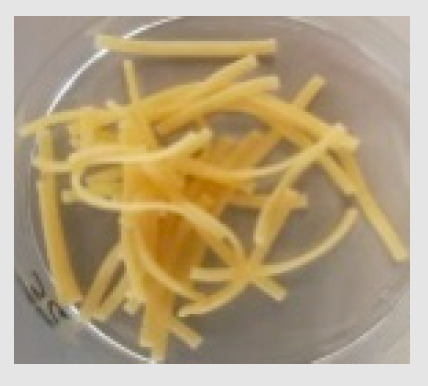	OCT: 4 min RIT: 0 min RRT: 15 min	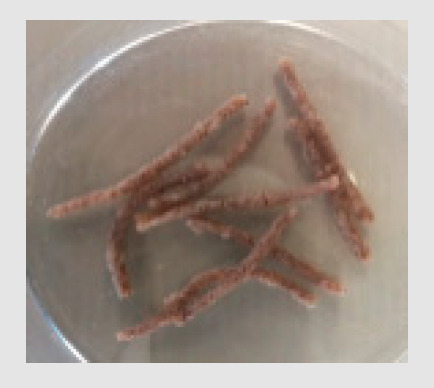	OCT: 8 min RIT: 15 min RRT: 0 min
Smooth	Evaluate the surface smoothness by looking at a pasta strand	Pasta S1 *		Pasta Integrale Barilla^®^	
		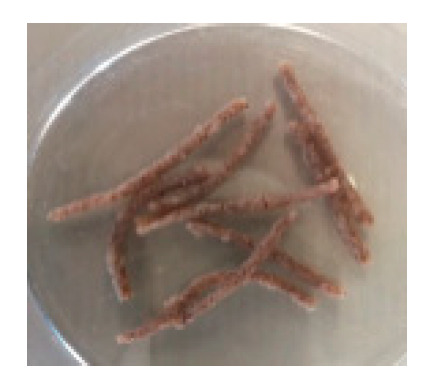	OCT: 8 min RIT: 15 min RRT: 0 min	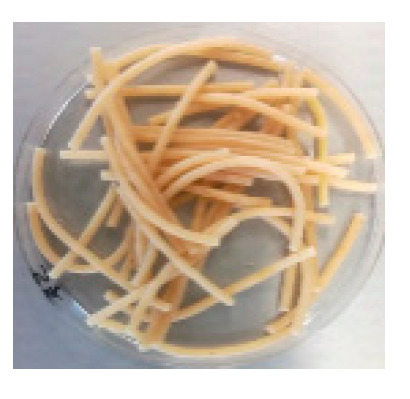	OCT: 8 min RIT: 0 min RRT: 30 min
Stickiness	Press a strand of pasta between your index finger and thumb. Evaluate how strong it sticks to your fingers	Pasta S8 ***		Pasta Integrale Barilla^®^	
		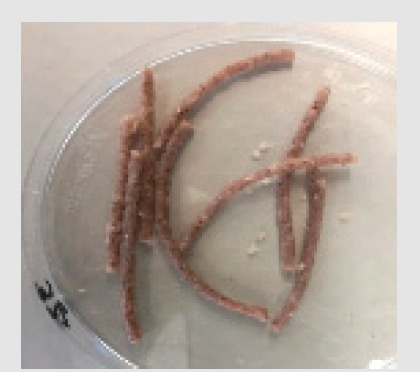	OCT: 7 min RIT: 0 min RRT: 0 min	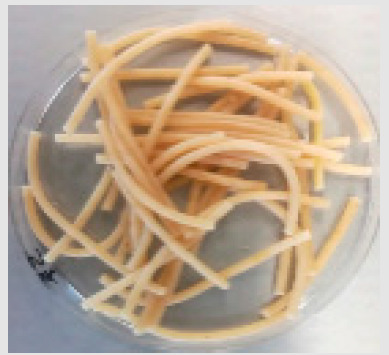	OCT: 8 min RIT: 0 min RRT: 30 min
Overall taste	Place a strand of pasta in your mouth and evaluate the overall taste intensity during mastication	Pasta *Mega di cato*^®^		Pasta S8 ***	
		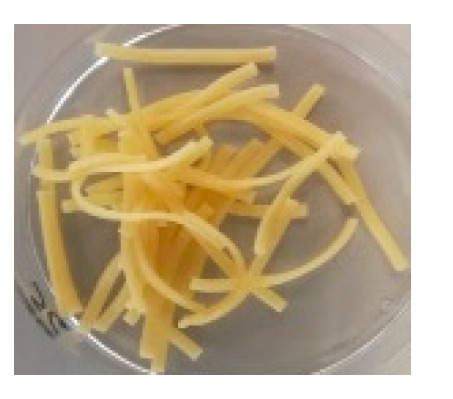	OCT: 4 min RIT: 0 min RRT: 15 min	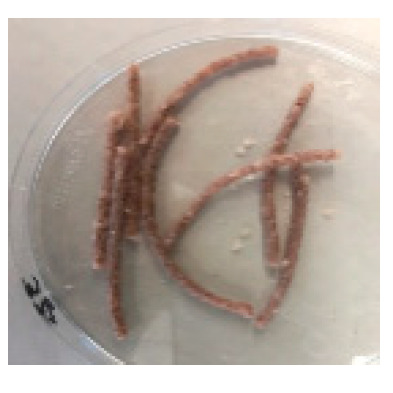	OCT: 7 min RIT: 0 min RRT: 0 min
Bitterness	Place a strand of pasta in your mouth and evaluate bitterness perception during mastication	Pasta *Mega di cato*^®^		Pasta S3 **	
		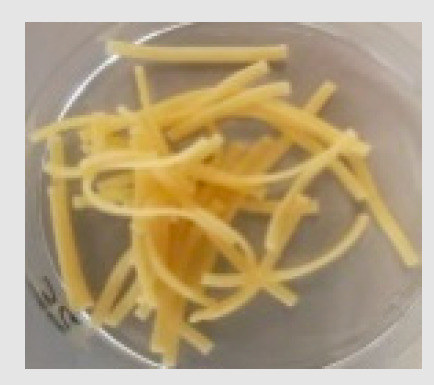	OCT: 4 min RIT: 0 min RRT: 15 min	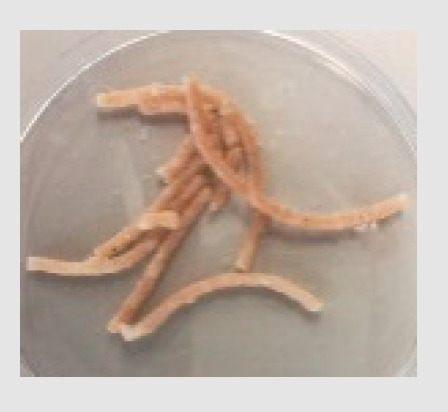	OCT: 7 min RIT: 2 min RRT: 5 min

OCT, optimal cooking time; RIT, rinsing time; RRT: Resting at room temperature prior to serving * Pasta S1: 79% buckwheat, 20% quinoa var. *negra collana*, 1% stabilizer; water content of dough, 60%; temperature profile, 87/98/100 °C; ** Pasta S3: 79% buckwheat, 20% quinoa var. *rosada taraco,* 1% stabilizer; water content of dough, 60%; temperature profile, 87/98/100 °C; *** Pasta S8: 79% buckwheat, 20% quinoa var. *kuchivila,* 1% stabilizer; water content of dough, 60%; temperature profile, 87/98/100 °C.

## Supplementary Materials

The following are available online at https://www.mdpi.com/2304-8158/9/12/1849/s1, Table S1: Raw data corresponding to pasta diameter, Table S2: Dataset corresponding to Figure 3, Table S3: Dataset corresponding to Figure 4, Table S4: Raw data corresponding to sensory evaluation.
